# *Ex vivo* Methods for Measuring Cardiac Muscle Mechanical Properties

**DOI:** 10.3389/fphys.2020.616996

**Published:** 2021-01-08

**Authors:** Walter E. Knight, Hadi R. Ali, Stephanie J. Nakano, Cortney E. Wilson, Lori A. Walker, Kathleen C. Woulfe

**Affiliations:** ^1^Department of Medicine, Division of Cardiology, University of Colorado Anschutz Medical Campus, Aurora, CO, United States; ^2^Department of Pediatrics, Division of Cardiology, Children’s Hospital, University of Colorado Anschutz Medical Campus, Aurora, CO, United States

**Keywords:** cardiac muscle, contractility and relaxation, mechanical function, cardiac physiology, myofilament

## Abstract

Cardiovascular disease continues to be the leading cause of morbidity and mortality in the United States and thousands of manuscripts each year are aimed at elucidating mechanisms underlying cardiac disease. The methods for quantifying cardiac performance are quite varied, with each technique assessing unique features of cardiac muscle mechanical properties. Accordingly, in this review, we discuss current *ex vivo* methods for quantifying cardiac muscle performance, highlighting what can be learned from each method, and how each technique can be used in conjunction to complement others for a more comprehensive understanding of cardiac function. Importantly, cardiac function can be assessed at several different levels, from the whole organ down to individual protein-protein interactions. Here, we take a reductionist view of methods that are commonly used to measure the distinct aspects of cardiac mechanical function, beginning with whole heart preparations and finishing with the *in vitro* motility assay. While each of the techniques are individually well-documented in the literature, there is a significant need for a comparison of the techniques, delineating the mechanical parameters that can are best measured with each technique, as well as the strengths and weaknesses inherent to each method. Additionally, we will consider complementary techniques and how these methods can be used in combination to improve our understanding of cardiac mechanical function. By presenting each of these methods, with their strengths and limitations, in a single manuscript, this review will assist cardiovascular biologists in understanding the existing literature on cardiac mechanical function, as well as designing future experiments.

## Introduction

The ultimate metric of cardiac function is its ability to pump blood to the entire body. There are numerous factors that contribute to this capability; therefore it is important to consider cardiac function from several different aspects. First, consider that the whole organ function depends on cardiac wall structures to maintain compliance to be able to stretch to accommodate blood entering the ventricle, synchrony of the contractile components to complete the complex and unique movement of the ventricle to optimally push blood into circulation, and a concerted relaxation period to allow filling. This whole organ function relies on cardiomyocyte function to exquisitely pair excitation-contraction coupling within a single cell and efficient propagation of signals to the next cardiomyocyte to work in concert. Finally, within the cardiomyocyte, the sarcomeric proteins interact precisely to develop force and relax. There are many systems providing regulation at each of these levels of complexity, from the cardiac conductance system regulating the depolarization and repolarization of the whole heart, to calcium handling systems within the cell, to z-disk proteins regulating sarcomeric stabilization and mechanotransduction. Given the absolute necessity for each of these regulatory systems to work in concert for an optimally functional heart, it is imperative when drawing conclusions from *ex vivo* methods of defining aspects of cardiac function to keep in mind what regulatory systems have been lost in the preparation and which remain intact. While *ex vivo* methods can introduce artificial scenarios, each can provide valuable insights into functional capabilities.

In this review we will consider the methodology for several commonly used *ex vivo* methods of assess cardiac function as well as the strengths and limitations of each. Finally, we will discuss how these techniques can be used in concert to complement and reinforce conclusions regarding cardiac function. It is important to note that there are several *in vivo* techniques for quantifying cardiac function in both humans and animal models. While these techniques, such as echocardiography, cardiac magnetic resonance imaging (MRI), computed tomography (CT), nuclear magnetic resonance (NMR), and positron emission tomography (PET) are critically important in assessing cardiac function *in vivo*, they are beyond the scope of this review. Here, we focus only on *ex vivo* techniques.

## Basic Concepts and Definitions

As mentioned above, there are many different facets of cardiac performance that define function from whole heart development of pressures down to molecular forces of the sarcomeric protein interactions. In order to ensure clarity and consistency in this review, we define parameters underlying cardiac compliance, systole, and diastole from the level of the whole heart to sarcomeric proteins (summarized in Table 1).

**TABLE 1:** Summary of basic mechanical parameters and the ability of each mechanical assay to accurately address each parameter.

Parameter	Langendorff/Working Heart	Trabeculae	Myocytes	Isolated Myofibrils
Compliance	Measurements = end diastolic pressure-volume relationship (EDPVR) and end diastolic volume (EDV). Contributions from a combination of ventricular stiffness and diastolic factors.	Measurement = passive tension. Contributions from ECM stiffness, residual myofibril cross-bridge formation, and titin isoform or modification.	Measurement = passive tension. Contributions from cytoskeletal protein stiffness, residual myofibril cross-bridge formation, and titin isoform or modification.	Measurement = passive tension. Contributions from residual myofibril cross-bridge formation and titin isoform or modification.
Systole	Force measurements = maximal apical tension and maximal left ventricular developed pressure (LVDP). Contributions largely from integrated myocyte tension generation Velocity measurements = maximal rate of tension development (dT/dt_*max*_) and maximal rate of pressure development (dP/dt_*max*_). Contributions are from integrated myocyte/myofibril activation kinetics as well as conduction system and structural components of the ventricle.	Force measurements = maximal force (F_*max*_). Contributions from many myocytes contracting together. Velocity measurements = typically measures tension redevelopment at steady state calcium (*k*_*TR*_). Contributions from rate of cross-bridge binding of many myocytes.	Force measurements = peak shortening and maximal force (F_*max*_). Contributions from many sarcomeres. Velocity measurements = rate of shortening, in skinned myocytes, typically measures tension redevelopment at steady state calcium (*k*_*TR*_). Contributions from rate of cross-bridge binding of many myocytes.	Force measurements = maximal force (F_*max*_). Contributions from a small bundle of sarcomeres. Velocity measurements = rate of activation (*k*_*ACT*_) and redevelopment at steady state calcium (*k*_*TR*_). Contributions from rate of cross-bridge binding of a small bundle of sarcomeres.
Diastole	Measurements = maximal rate of tension loss (dT/dt_*min*_) and maximal rate of pressure loss (dP/dt_*min*_). Contributions from integrated myocyte relaxation kinetics, ventricular stiffness, and conduction system.	Measurements = monophasic relaxation kinetics. Contributions from ECM composition, and myosin cross bridge dissolution (off-rate).	Measurements = rate of relengthening, in skinned myocytes N.A. Contributions from cellular compliance and myosin cross bridge dissolution.	Measurements = linear and exponential relaxation duration and rate. Contributions from as small bundle of sarcomeres

*N.A, Not applicable; ECM, extracellular matrix; PTMs, post-translational modifications.*

### Compliance

The compliance of the ventricular wall is highly important as it dictates the volume of blood that enters the ventricle during diastole and is strongly related to the development of the necessary pressures for the heart to pump blood to the body. Generally, compliance is often thought of in conjunction with ventricular stiffness: a heart with increased stiffness will have decreased compliance. At the whole organ level, there are many factors that contribute to compliance of the ventricular wall, including extracellular matrix (ECM) as well as stiffness of the cardiomyocytes themselves. Mechanically, compliance of the ventricular wall can be thought of as passive tension, which is the tension developed in a muscle as it lengthens (Granzier and Wang, 1993). When thinking of the ventricular wall, as blood fills the ventricle during diastole, the wall is lengthening to accommodate the incoming blood. This expansion of the chamber results in tension that is separate from the active tension generated during contraction.

Overall, the two main components central to passive tension are collagen and titin, with intermediate filaments, and/or microtubules providing roughly 10% of the passive tension (Granzier and Irving, 1995). Collagen is a predominant component of the ECM and imparts structural integrity to the ventricle. Titin is a spring-like structural protein integral to the sarcomere, which attaches the M line to the z disk (Granzier and Labeit, 2004). At physiologic sarcomere lengths, as length increases (as with ventricular filling), passive tension contributed by titin increases exponentially (Granzier and Irving, 1995). Furthermore, titin plays a primary role in length-dependent activation and provides the majority of passive tension at physiologic sarcomere lengths (∼1.9–2.2 μm) (Fukuda et al., 2003; Chung and Granzier, 2011). In contrast, collagen plays a role in the elastic force of the ventricle extracellularly and contributes more to passive tension at longer sarcomeric lengths (or greater volumes) (Granzier and Irving, 1995; Chung and Granzier, 2011). When considered at the level of the myofilament, passive tension is an integral property of the sarcomere which regulates other mechanical properties of the sarcomere. For example, passive tension is also generated by viscosity within the sarcomere and this element limits the velocity of shortening (de Tombe and ter Keurs, 1992).

Given that several different factors contribute to passive tension, it is important to consider the structures present in each method. For example, the passive tension of a whole heart or a multicellular preparation will have contributions from extracellular matrix (ECM), as well as cytoskeletal (intermediate filaments and microtubules) and sarcomeric (titin) structures. However, a single cell, cell fragment, or myofibril preparation will have contributions solely from cytoskeletal and/or sarcomeric structures.

In *ex vivo* whole heart preparations, several measurements can be used to define passive tension and ventricular compliance changes. These measurements include the end diastolic pressure-volume relationship (EDPVR) and end diastolic volume (EDV). Passive tension can also be measured in trabecular, whole cell, and myofibril *ex vivo* methods.

### Systole

One of the most easily understood aspect of cardiac function is contraction during systole where the ventricle performs a synchronized development of force, thereby decreasing the ventricular chamber volume in such a way to push blood out to the circulatory system. If this contraction is decreased, the results are apparent, often catastrophic, and are evidenced by decreased cardiac output; therefore, when studying the physiology of different cardiac diseases and mutations, an immediate functional measurement or output relays information about contraction.

Contractility is the ability of a myocyte or muscle fiber to shorten and develop force (Muir and Hamlin, 2020). Underlying actin and myosin interactions dictate the development of force as well defined in the sliding filament and the cross bridge theories (Huxley and Niedergerke, 1954; Huxley and Simmons, 1971). Based on these accepted theories, at physiologic sarcomere lengths, the number of actin-myosin cross-bridges that are cycling are proportional to the force generated (Regnier et al., 2004). In addition, the force that a muscle fiber can produce is related to the average sarcomere length within that fiber, suggesting that force generation is proportional to the overlap between actin and myosin (Gordon et al., 1966). Lastly, contractility can be altered by changes in calcium sensitivity and cooperativity, two specific attributes of the sarcomeric protein interactions.

Calcium sensitivity is a measurement of the myofilament’s sensitivity to calcium. Calcium regulation of the thin filament in cardiac muscle dictates force generation through calcium binding to troponin C resulting in a conformational change within the troponin-tropomyosin complex to expose actin binding sites and allowing actin-myosin cross bridge formation to proceed (Kobayashi and Solaro, 2005). Cooperativity also impacts myofilament force generation and refers to the effects of one protein on the function of another. For example, increased cross-bridge formation impacts calcium binding to TnC leading to increased affinity of TnC for calcium (Grabarek et al., 1983; Martyn et al., 2001; Wang and Kerrick, 2002). Although the sarcomeric protein interactions and resulting force development are key factors underlying contractility, other cellular factors play critical roles in contractility. Although not covered in this review, calcium dynamics within the individual myocytes and synchronous depolarization, regulated by the conduction system, are critical in the coordinated contractile function of the heart.

An important consideration when understanding contractility is load. Physiologically, the contractility/force an intact cardiomyocyte generates within the heart is directly related to preload, such that an increase in preload results in stretching of the cell, increasing the sarcomere length and thus passive tension. The Frank-Starling Law dictates that increases in preload result in an increase in active tension (Kameyama et al., 1998). Accordingly, when studying active tension *in vitro* it is important to recognize that the majority of experiments are conducted on “unloaded” tissue/cells and that sarcomere length is experimentally set at the time of force measurement.

In whole heart preparations, there are several measurements that evaluate systolic function including left ventricular systolic pressure (LVDP), fractional shortening, and the rate of pressure development (dP/dT_*max*_). At the level of the cardiomyocyte and subcellularly, several measurements of force generation are reported. The first, F_*max*_, is the total tension generated at maximal calcium concentration. F_*max*_ is a measure that includes both active tension and passive tension; therefore, the aforementioned contributors to passive tension as well as active tension should be considered. The second parameter important for understanding active tension is the rate of tension development (*k*_*ACT*_), which describes the rate of sarcomeric shortening. In addition, k_*TR*_ is used to define the rate of tension redevelopment, which is performed by rapid release-restretch of cardiac preparations at steady-state calcium, allowing analysis of both cross-bridge detachment and attachment. Thus, assuming a two-state cross-bridge model, *k*_*TR*_ is a direct measurement of the sum of cross-bridge attachment and detachment kinetics (Wang and Kawai, 2013).

### Diastole

Given the pump function of the heart, it is important for the ventricle to relax efficiently following systole to allow a sufficient volume of blood to fill the ventricle before the next contraction. Naturally, relaxation parameters are intrinsically related to parameters of contractility with calcium handling (uptake and removal) and conduction synchrony integral to the process. Relaxation at the level of the sarcomere is dictated by an overall decrease in the number of bound cross-bridges in response to removal of calcium. Furthermore, it is important to note that aspects of diastole and relaxation are also closely related to compliance of the ventricular wall and also passive tension. If the wall is stiffened due to increased deposition of ECM leading to decreased compliance of the ventricle, this in turn impacts diastole wherein the amount of blood that can enter the ventricle is decreased.

In whole heart preparations, the rate of pressure loss (dP/dT_*min*_) is a measurement that evaluates diastole. Furthermore, in methods evaluating cellular relaxation, rate and time for cell relengthening can suggest differences in relaxation. Subcellular techniques take advantage of the fact that the small size of the preparation allows for rapid calcium removal away from the sarcomere, allowing quantification of the kinetics of the thin filament inactivation (in theory) and cross bridge “off-rate” kinetics (Poggesi et al., 2005). In these small subcellular preparations, actin and myosin binding and release most closely determine relaxation parameters (Poggesi et al., 2005; Vitale et al., 2019). It is also important to consider that different properties of the sarcomere, as well as external stresses, may also alter relaxation kinetics (Brutsaert and Sys, 1989; Poggesi et al., 2005).

## Methods

**FIGURE 1:**
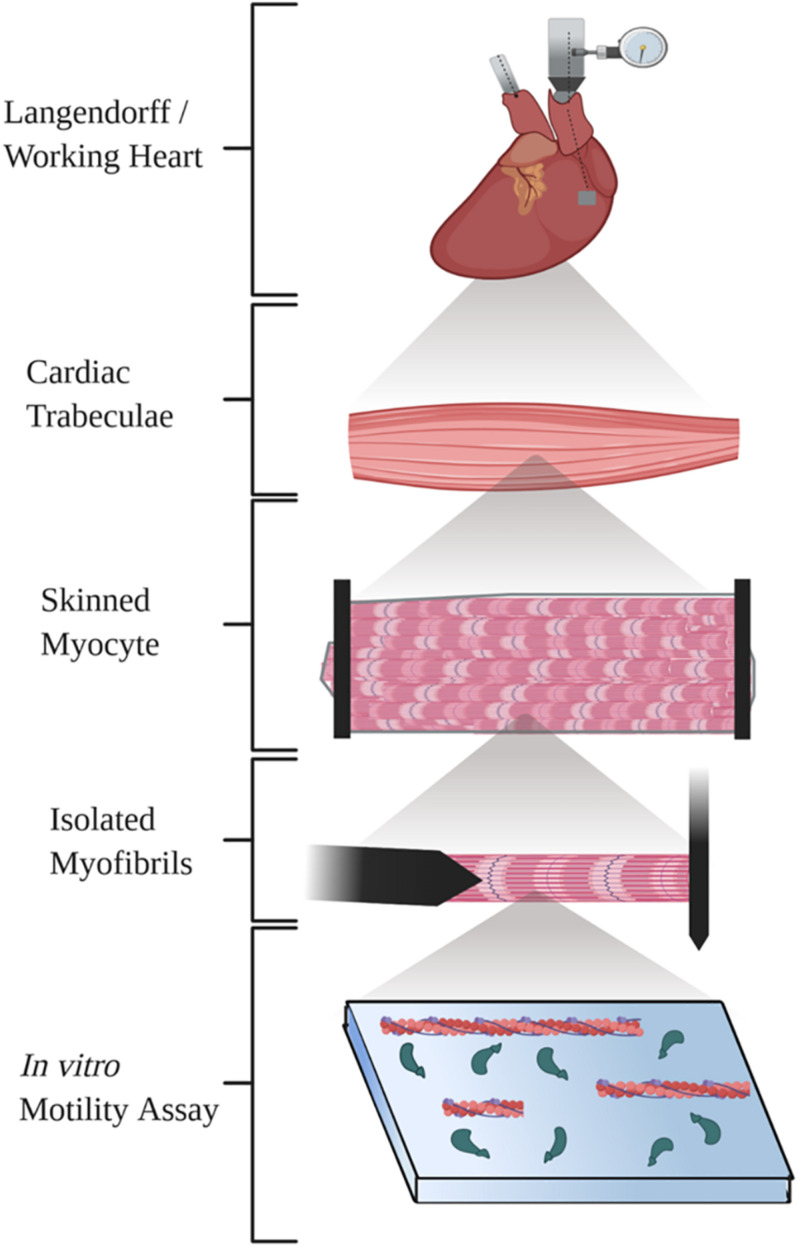
Diagram illustrating the five techniques summarized in the review. Created with BioRender.com.

Here, we review five *ex vivo* methods commonly used to study cardiac mechanics (Figure 1). We briefly describe a basic methodologic overview for each technique and provide several references that will allow researchers to find detailed methods, discuss the functional parameters and benefits of the technique, and finally examine important considerations to keep in mind with each technique.

### Whole Heart Techniques

Two techniques (Langendorff and working heart) are often used to characterize whole heart function *ex vivo*.

#### Basic Methods of the Langendorff Heart

In this technique, the beating heart is rapidly excised and hung by the aorta on a perfusion system. This provides retrograde perfusion through the aorta which closes the aortic leaflets, allowing the perfusate to supply nutrients and oxygen to cardiomyocytes through the coronary circulation. Under these circumstances, the heart continues to beat spontaneously, and contractility is measured using either a pressure transducer attached to the cardiac apex or a balloon transducer inserted into the left ventricle (LV). The pressure transducer allows direct measurement of tension, in terms of peak systolic tension, diastolic tension, as well as the change in tension over time (dT/dt) (Langendorff, 1895; Sutherland and Hearse, 2000; Headrick et al., 2001; Liao et al., 2012). If the transducer is attached to a balloon inserted into the LV, similar parameters of pressure can be measured (left ventricular diastolic pressure (LVDP), maximal and minimal dP/dt), in addition to interrogating of the LV pressure-volume relationship (Kameyama et al., 1998) through progressive inflation of the balloon to different volumes. Further, the Langendorff heart can be externally stimulated to study force-frequency (Ng, A, Grupp et al., 1991) relationships.

#### Basic Methods in Working Heart Preparation

The second technique, the working heart preparation, is an extension of the Langendorff method (Salama and Hwang, 2009), however, in the working heart preparation, the left atrium is directly cannulated and perfusate flows anterograde. This allows the heart to “work” by pumping fluid into the aorta and downstream tubing, typically into a pressure chamber that mimics vascular resistance (Neely et al., 1967; Grupp et al., 1993). As with the Langendorff method, working heart method can be externally stimulated or allowed to contract at its endogenous pace-making rate. In addition to the various parameters that can be determined in the Langendorff heart, the working heart also enables quantification of LV ejection volume, aortic pressure, stroke volume, cardiac output, work, and power (Ng, A, Grupp et al., 1991; Grupp et al., 1993; Gauthier et al., 1998; Pedersen et al., 2018). Importantly, in the working heart, both preload and afterload can be dynamically modulated (Neely et al., 1967; Gauthier et al., 1998; DeWitt et al., 2016) through experimental manipulation of flow rate and resistance.

The *ex vivo* whole heart preparations offer several benefits for studying both physiologic and pathologic cardiac function. Each experimental set-up can use a flexible variety of perfusion buffers or even whole blood, allowing assessment of whole-organ response to different metabolites, drugs, or changes in calcium concentration (Takeuchi et al., 1999; Sutherland and Hearse, 2000; Skrzypiec-Spring et al., 2007; Liao et al., 2012). Importantly, contractile activity in these whole-heart experiments is quantified in a preparation containing all cell types (cardiomyocytes, endothelial cells, fibroblasts, inflammatory cells and others) and with the structures of the heart completely intact (including the conduction system). This allows assessment of whole organ dynamic adaptations to alterations in hemodynamic load, heart rate changes, and/or exogenous substances delivered via perfusion. Many of the contractile parameters that can be measured in these preparations cannot otherwise be measured without using *in vivo* approaches. However, in contrast to *in vivo* studies, the Langendorff and working heart preparations are more amenable to experimental manipulation. For example, while the acute effects of both pressure or volume overload on contractility can be interrogated using these systems; these effects cannot be easily studied *in vivo*. Paracrine signaling is also preserved in these preparations, which means modulation of cardiomyocyte contractile function by fibroblasts and endothelial cells can be assessed. As drugs introduced into coronary perfusate will rapidly diffuse throughout the coronary vasculature, both preparation types are also ideal systems for studying acute cardiac response to drugs, chemicals, and catecholamines (Fukuda et al., 1989; Grupp et al., 1993; DeWitt et al., 2016). Advanced optical techniques can be combined with the Langendorff or working heart setups to allow real-time measurement of Ca^2+^ flux, metabolite production, or oxygen use (Salama and Hwang, 2009; Liao et al., 2012; Venkataraman et al., 2012; Kuzmiak-Glancy et al., 2015). Both the Langendorff and working heart setups allow assessment of the acute effects of cardiac ischemia-reperfusion injury (I/R) in an *ex vivo* system. Ischemia can either be introduced globally (by shutting off inflow of perfusate) or regionally (by ligating a coronary artery) (Skrzypiec-Spring et al., 2007). Unlike *in vivo* I/R injury, the effects of both ischemia and reperfusion on contractility can be monitored continuously, and in real time (Headrick et al., 2001).

#### Important Considerations

These experiments are technically challenging which introduces the concern about comparing differences across laboratories. Pressure catheters must be precisely placed for the collection of physiologically relevant data (Schechter et al., 2014). Improperly sized or inflated LV balloons can lead to inaccurate contractile data; for example, undersized balloons result in reduced measures of contractility and pressure (Headrick et al., 2001). Similarly, variations in temperature can profoundly affect myocardial contractility. Cardiac function decreases quickly at temperatures below 35°C, while temperatures in excess of 39°C can cause cardiac damage (Sutherland and Hearse, 2000; Headrick et al., 2001). Changes in pacing rates, perfusion flow rates, oxygenation, composition, temperature, and, if applicable, LV balloon dynamics and afterload level can all introduce inter-experimental variability, potentially leading to discordant results. Given all of these potential sources of variability, comparison of data acquired from different Langendorff or working heart setups may be challenging, thus making use of proper experimental controls crucial.

### Multicellular Techniques

#### Basic Methods of the Multicellular Preparations

Trabeculae and papillary muscles are typically employed as proxies for the myocardium in the investigation of cardiac ventricular mechanics as they are linearly arranged, present throughout both ventricles of the heart, and are thought to be roughly homologous with the ventricular free walls and septum (Goo et al., 2009), however, small ventricular wall strips have also been used (Fatemifar et al., 2018). It is important to describe the origin of the muscle tissue (papillary, trabecular or free wall) as it has recently been shown that trabeculae are stiffer than either papillary muscles or myocardial strips from the same heart (Fatemifar et al., 2018), and can contain more collagen. Additionally, RV tissues have been shown to be stiffer than LV tissues (Sacks and Chuong, 1993; Fatemifar et al., 2018). Here, we will review the basic experimental methods for isolation and interrogation of trabeculae; nevertheless, the same experimental considerations apply to each multicellular preparation. To undertake mechanical measurements, thin, uniform, and unbranched trabeculae are rapidly excised from perfused hearts. Importantly, while this technique can be performed on tissue isolated from either ventricle, the majority of the published studies describing multicellular methods have been completed in trabeculae or papillary muscles isolated from the right ventricle (Fatemifar et al., 2018). The excised tissue includes a portion of ventricular free wall tissue on one end and a portion of the AV ring and tricuspid valve or ventricular free wall on the other, which provides structure for metal hooks and/or rings to attach the trabeculae to a motor on one side and a force transducer on the other (Krueger and Pollack, 1975). Thin trabeculae are chosen to reduce core hypoxia and ensure adequate diffusion (Goo et al., 2009). Isolated trabeculae are stretched in the presence of extracellular calcium until sarcomeres are at a specific length. This is a critical consideration since different sarcomere lengths can yield varying conclusions regarding force generation (Rodriguez et al., 1992). The trabeculae can be iontophoretically loaded with a calcium indicator for measurements of intracellular calcium (Gao et al., 1994) or conversely, studies can be conducted on skinned trabeculae which involves permeating (skinning) the sarcolemmal membrane (Kentish et al., 1986; Gwathmey and Hajjar, 1990; Gao et al., 1994) for measurement of sarcomeric mechanics at fixed calcium levels. The skinning process consists of incubating tissue in a solution with a non-ionic detergent to disrupt the sarcolemmal barrier.

Multicellular preparations are important models for assessing the contributions of the ECM in mechanical measurements. This is crucial in disease models of myocardial infarction, cardiac hypertrophy, and heart failure where changes in the ECM may potentially contribute significantly to changes in cardiac performance. Of particular importance, similar to whole-heart preparations, multicellular preparations can be used to assess function where multiple cell types essential to contractile function in the heart remain in place; thereby allowing assessment of how various alterations in proteins impact mechanical function in a multicellular system. In addition, the use of computer programs has enabled assessment of how acute volume and load changes impact function in multicellular preparations through computational modeling. Using this type of modeling, researchers can combine computational modeling with the multicellular systems to interpret physiologic and pathologic functional changes in response to volume and load changes (De Tombe and Little, 1994; Britton et al., 2017; Guidry et al., 2020).

#### Important Considerations

As the muscles are attached using clips or hooks, there is the possibility of internal sarcomere shortening in response to damage (ter Keurs et al., 1980; Garnier, 1994). Further, because trabeculae are often used as a surrogate for intact ventricular myocardium, an inherent assumption in their use is that these trabeculae preparations are homologous with the free wall (Goo et al., 2009; Serrani, 2014). The most important distinction between trabeculae and compact myocardium is the orientation of the cardiac fibers in each of these tissues. The muscle fibers of intact myocardium are oriented in a spiral, while the fibers of trabeculae are oriented linearly along the axis of the trabeculae (Goo et al., 2009; Serrani, 2014). It is important to consider fiber orientation differences when applying conclusions derived from trabeculae to free ventricular wall (Serrani, 2014).

### Single Myocyte Techniques

Similar to studies in multicellular cardiac preparations, single myocyte preparations inherently preserve many of the geometric and mechanical constraints of the myofibrillar apparatus present *in vivo*. Importantly, the three-dimensional sarcomeric structure is essential to consider in mechanical studies since this geometry both imposes limits on, and facilitates contractile protein interactions (Gordon et al., 2000). Isolation of single cells or cell fragments allows for detailed characterization of myocyte contractile properties without heterogeneity (in orientation, strain, and force generation) and without influence of surrounding extracellular matrix proteins present in multicellular preparations. Additionally, as with whole-heart and multicellular preparations, the effect of preload on myofilament tension generation (length-tension relationship) can be investigated directly by manipulation of the sarcomere length.

#### Basic Methods of the *in vitro* Intact Myocyte Isolation

Cardiac myocytes are routinely isolated by rapidly excising the heart and performing retrograde-perfusion with an enzymatic buffer. After isolation, cells can be loaded with Fura-2 or other calcium-sensitive dyes, and whole cell contractility and calcium cycling can be analyzed using edge detection- and fluorescence-microscopy (Jeong et al., 2010). With the use of additional or alternate fluorescent dyes, it is possible to quantify other intracellular parameters such as metabolic state during each phase of contraction. Isolated cardiomyocytes are placed in a flow chamber and mounted on the stage of an inverted microscope. Fourier transformation of the cell image tracks sarcomere length changes during paced contraction to determine cell shortening, and a photomultiplier is used to measure the ratio of Fura-2 fluorescence (excited at 340 and 380 nm) to obtain intracellular calcium (Jeong et al., 2010). From the sarcomere length record it is possible measure peak shortening, rate of contraction at half peak twitch force, rate of relaxation at half peak twitch force, duration of contraction at half peak twitch force and force-time integral (area under the curve). From the Fura-2 fluorescence measurements of peak calcium, rate of rise at half peak calcium, rate of fall at half peak calcium during relaxation, duration of transient at half peak calcium, and the total calcium released in a transient (area under the curve) can be obtained. Measurements can be carried out at pacing frequencies that range from 1 Hz to greater than 5 Hz (the choice of pacing frequency is dependent on the species of animal the cardiomyocytes were isolated from) to permit the construction of force-frequency curves. Further, as with other techniques assessing functional parameters, another important consideration is the temperature at which the measurements are taken.

This technique enables assessment of myocyte shortening (a surrogate for contractility) in an intact cardiomyocyte without the impact of the ECM, allowing for measurement of changes in function of the sarcomeric machinery with intact calcium handling.

#### Important Considerations

Isolation of cardiomyocytes can be difficult, especially from mice, and the surviving cells may not be representative of all cells in the heart. Additionally, enzymatic isolation results in loss of cell-to-cell connections and may damage extracellular receptors, limiting the ability to assess cell signaling pathways. Furthermore, cells are paced at a rate far below the physiologic rate in rodents and this may introduce non-physiologic variables. Lastly, while shortening is surrogate measure of contractility, it is important to remember that intact isolated cell preparations are unloaded and the results cannot be directly compared to multicellular and whole-heart preparations as the sarcomere lengths are shorter than *in vivo*. Further, since many parameters are dependent on sarcomere length (i.e., calcium sensitivity and length-dependent activation), it is important to recognize these experiments do not represent physiological conditions in this respect.

#### Basic Methods of Skinned Myocytes

Though myocardial contractility is influenced by both intracellular calcium availability and alterations in myofilament calcium responsiveness (Endoh and Blinks, 1988), mechanical studies of permeabilized (skinned) preparations highlight the direct effects of calcium on the myofilament (Fabiato and Fabiato, 1975). Skinned myocyte preparations, in particular, permit effective external control of the intracellular environment (Perreault et al., 1990). Similar to the skinning process in multicellular preparations, myocytes’ sarcolemmal barriers are disrupted by non-ionic detergents in order to assess calcium concentration on myofilament tension generation. Importantly, skinning also allows for exchange or partial replacement of thin filament proteins to examine roles of protein isoforms or mutations (Gordon et al., 2000). Importantly, skinned cardiomyocytes can be isolated from frozen biopsies, providing more flexibility in experimental design and collection of tissues. Following skinning and isolation, a single, rod-shaped myocyte or myocyte fragment with clear striations is positioned horizontally between two pins; one pin is attached to a force transducer and the other to a motor. The ends of the myocyte are glued to the tips of the pins and the myocyte is lifted and stretched to the desired sarcomere length. The myocyte is then exposed to several different solutions with known calcium concentrations and the tension generated and rate of force redevelopment (*k*_*TR*_) can be measured. From these measurements, calcium sensitivity and length dependent activation can be assessed. This technique allows assessment of the direct effects of calcium on myocyte contractility. Furthermore, skinned myocytes measurements also allow quantification of passive stiffness and cooperativity and provides for direct interrogation of the effects of pharmacologic agents and recombinant proteins on both passive and active contractile properties of the cardiomyocyte.

#### Important Considerations

A unique technical hurdle to the assessment of mechanics in skinned single myocytes arises from the need to glue the ends of the cell to the force transducer and motor pins with sufficient strength, yet without damaging the myocyte (Brady, 1991). Limitations also arise from both the single myocyte preparation and the skinning process. First, alterations in the extracellular matrix or cell-to-cell connections (i.e., desmosomes) play large roles in development of a cardiomyopathy phenotype, yet are unlikely to be present in single cell preparations (Sequeira et al., 2014). Second, it has been noted previously that single myocytes that survive the isolation procedure may not be representative of the entire heart (De Tombe, 1998). Thus, skinned single cell preparations are not intended to represent a completely physiologic state but are a useful tool to delineate changes in calcium sensitivity, active tension, and rate of force development.

### Skinned Myofibril Technique

#### Basic Myofibril Technique

For these experiments, very small bundles of sarcomeres (myofibrils) are isolated from either frozen tissue or cultured cardiomyocytes using mechanical disruption in a solution with a non-ionic detergent, and attached using electrostatic interactions to two glass microtools (Poggesi et al., 2005; Woulfe et al., 2019). One microtool is a cantilevered force probe with a known compliance, while the second microtool is attached to a piezo motor for length control. Isolated myofibrils are demembranated and while the sarcomeric proteins maintain their crystalline structure, sarcoplasmic reticulum and mitochondria are not functional. This allows for the control of ATP, phosphate, and calcium concentrations in a nearly diffusion-independent manner. Currently, two methods are used for altering the solutions to which the myofibrils are exposed. First is the fast-switch perfusion method wherein the myofibril is exposed to different solutions from a double-channel micropipette capable of very small, rapid movements (Colomo et al., 1997). Alternatively, the myofibrils can be exposed to different solutions through stop or quench flow (Barman et al., 1998). Due to the small nature of the myofibril preparation, this technique serves to allow quantification of both passive and active tension, the rate of force development, calcium sensitivity, and is ideally suited for quantifying relaxation kinetics.

Since myofibrils have a very small diameter, this permits rapid equilibration of solutions allowing analysis of kinetics of the sarcomeric proteins without influence of diffusion (Poggesi et al., 2005; Stehle et al., 2009). Furthermore, since the sarcomeric proteins are intact and interacting within full sarcomeres, this technique enables accurate measurement of sarcomere length, and extremely high resolution measurement of relaxation kinetics. Additionally, there is no glue necessary to attach the myofibrils to the microtools which removes the impact of adhesive compliance on myofibril kinetics. Finally, this technique also allows exchange of thin filament accessory proteins to determine how isolated mutations alter function (Piroddi et al., 2019; Lin et al., 2020).

#### Important Considerations

The methods to skin the myofibrils remove regulatory proteins and it is likely that some of the viscoelastic forces are lost by isolating sarcomeres (Stoecker et al., 2009). Further, only “healthy” myofibrils are measured by this technique, as highly damaged myofibrils cannot contract and therefore will not produce data. If one is considering a condition that leads to damaged or weakened sarcomeric structures, then this method by necessity self-selects for the intact or heathiest ones. Another important caveat is that cross-bridge detachment is not directly equivalent to muscle relaxation. This is because calcium removal is a key factor this process and a skinned myofibril lacks that regulatory unit (Little et al., 2012; Chung et al., 2016).

### *In vitro* Motility Technique

With many different complex molecular interactions occurring within the sarcomere, there are a range of different methods have been designed to answer specific questions. In general, the approach taken is to reduce the number of proteins in technique to isolate particular aspects of function. For clarity, we will focus on one such method that specifically addresses interactions between the thin filament and the thick filament in isolation.

#### Basic Methods of the *in vitro* Motility Assay

*In vitro* motility assays utilize isolated subfragments of myosin (typically the myosin head and some length of the neck) adhered to a surface and isolated thin filaments or actin alone in order to measure velocity and force at different calcium concentrations (Sheetz and Spudich, 1983; Spudich et al., 1985). It is important to recognize that while we are not discussing other types of single-molecule preparations of isolated sarcomeric proteins, some of the same considerations can be applied to single molecule preparations in general, and as with the other preparations we review here, all conclusions need to consider assumptions inherent to each experiment.

The *in vitro* motility assay is ideal for providing reductionist data with regard to active tension development, calcium sensitivity, and cooperativity. The tension that can be generated and velocity of the actin or thin filament movement along immobilized myosin fragments reflects the efficiency of the cross-bridge cycling and can highlight differences in actin-myosin interactions (Liang et al., 2003). As this preparation simplifies sarcomeric protein interactions, one of its key advantages is that it allows researchers to delve into basic functional questions. For example, *in vitro* motility assays have been extremely important in defining actin-myosin interactions and dissecting complex concepts of cooperativity (Albet-Torres et al., 2009).

#### Important Considerations

When extrapolating findings from *in vitro* motility assays to cell and whole organ function, some caution needs to be taken. The loss of accessory proteins as well as loss of integral sarcomeric structure may have significant impact on sarcomeric function, such that while conclusions regarding specific subcellular interactions can be drawn, extending these concepts to the broader whole-cell or whole-organ function may not be wholly accurate. Other limitations include technical variations in orientation of the myosin heads, the composition of the surface to which the heads are adhered, and how the solution composition and temperature impact function (Mansson et al., 2018).

## Discussion

### How to Use These Methods to Complement/Answer Questions

Here we have briefly provided an overview of common *ex vivo* techniques used to quantify cardiac performance from the level of the whole organ function to isolated protein-protein interactions. Each of these techniques has strengths and weaknesses that have been highlighted. No *ex vivo* single method can completely define physiologic or pathophysiologic cardiac performance and still allow for molecular interrogation of such changes. However, when used in conjunction, researchers can use these methods to begin to identify organ-level disruptions to function and the molecular cause of such disruption. For example, by employing techniques that maintain intact multicellular interactions (either whole heart or isolated muscle preparations) in conjunction with skinned cardiomyocyte preparations, researchers can gain important information regarding abnormalities in the calcium handling properties of the muscle. In another example, consider investigating the effects of a novel, potential HCM-causing mutation in a sarcomeric protein found in a human heart explant. Initially, the effects of this mutation on myofibril mechanics and/or skinned cardiomyocytes from this heart explant could be assessed to determine how the mutation impacts force production, calcium sensitivity of the myofilament, and cooperativity. If this mutation is in actin or myosin, sliding filament assays could be performed on mutant protein. A mouse harboring this mutation could then be generated, and the impact of the mutation on organ-level performance could be assessed by the Langendorff/working heart (in addition to the various *in vivo* techniques which are not discussed here) as well as intact single cell measurements. Taken together, these studies would lead to considerable understanding of the molecular effects of a mutation on the sarcomere, its effects on myofibrils, cardiomyocytes, and ultimately the heart. Skinned cardiomyocytes and myofibrils allow direct analysis of protein mutations and provide platforms for *in vitro* assessment of therapeutic interventions. Together, assessment of cardiac contractile function at each level can provide important functional information accelerating understanding of cardiovascular disease. In recent years, single myocyte and myofibril preparations have become more frequently used, in part, because multicellular preparations have demonstrated (i) heterogeneity of action potentials and unpredictable electrical activity of the various cell types in multicellular preparations, (ii) extracellular spaces that limit diffusion and lead to the accumulation or depletion of ions, (iii) a lack of uniform cellular orientation in the multicellular preparation, (iv) a lack of uniform sarcomere shortening during isometric contraction, and (v) an influence of the ECM (non-contractile proteins) on muscle contraction (Krueger and Pollack, 1975; Noble, 1976; Eisenberg, 1980; Watanabe et al., 1983; Brutsaert, 1987; Ohayon and Chadwick, 1988; Garnier, 1994). However, it is important to recognize and acknowledge the limitations inherent in these techniques in order to avoid overstating conclusions. Overall, these techniques all provide important insights into cardiovascular function and allow researchers to uniquely dissect cardiac function.

## Conclusion

As highlighted throughout this review, cardiac mechanical function is an important component of overall cardiovascular physiology, and can be assessed by a diverse array of experimental techniques. We have summarized five common *ex vivo* techniques used by researchers worldwide. Each of the techniques described has a number of strengths and weaknesses, yet when used in combination, they can provide a comprehensive understanding of the consequences of perturbations in cardiovascular function. While all of these methods may be used alone in set of studies, together, or combined with other experimental techniques, they represent a powerful set of tools available to cardiovascular researchers.

## Author Contributions

All authors wrote and edited the manuscript. WK designed the figure. LW and KW conceptualized the topic.

## Conflict of Interest

The authors declare that the research was conducted in the absence of any commercial or financial relationships that could be construed as a potential conflict of interest.
